# Impact of prostheses on quality of life and functional status of transfemoral amputees in Tanzania

**DOI:** 10.4102/ajod.v10i0.839

**Published:** 2021-09-07

**Authors:** Ericka P. von Kaeppler, Alexander Hetherington, Claire A. Donnelley, Syed H. Ali, Corin Shirley, Sravya T. Challa, Emily Lutyens, Billy T. Haonga, Saam Morshed, Jan Andrysek, David W. Shearer

**Affiliations:** 1 Institute of Global Orthopaedics and Traumatology, University of California San Francisco, San Francisco, United States of America; 2 LegWorks, Inc., Buffalo, United States of America; 3 Department of Orthopaedic Surgery, Muhimbili Orthopaedic Institute, Dar es Salaam, Tanzania; 4 Bloorview Research Institute, Holland Bloorview Kids Rehabilitation Hospital, Toronto, Canada

**Keywords:** low- and middle-income countries, transfemoral amputation, above-knee prosthesis, quality of life, functional status, Tanzania

## Abstract

**Background:**

The rise of diabetes and traumatic injury has increased limb loss-related morbidity in low- and middle-income countries (LMICs). Despite this, the majority of amputees in LMICs have no access to prosthetic devices, and the magnitude of prosthesis impact on quality of life (QOL ) and function has not been quantified.

**Objectives:**

Quantify the impact of prostheses on QOL and function in Tanzanian transfemoral amputees.

**Method:**

A prospective cohort study was conducted. Transfemoral amputees at Muhimbili Orthopaedic Institute were assessed twice before and three times after prosthetic fitting using EuroQol-5D-3L (EQ-5D-3L), Prosthetic Limb Users Survey of Mobility (PLUS-M), 2-minute walk test (2MWT) and Physiologic Cost Index (PCI). Data were analysed for change over time. Subgroup analysis was performed for amputation aetiology (vascular or non-vascular) and prosthesis use.

**Results:**

Amongst 30 patients, EQ-5D, PLUS-M and 2MWT improved after prosthesis provision (*p <* 0.001). EuroQol-5D increased from 0.48 to 0.85 at 1 year (*p* < 0.001). EuroQol-5D and 2MWT were higher in non-vascular subgroup (*p* < 0.030). At 1-year, 84% of non-vascular and 44% of vascular subgroups reported using their prosthesis (*p* = 0.068).

**Conclusion:**

Prosthesis provision to transfemoral amputees in an LMIC improved QOL and function. This benefit was greater for non-vascular amputation aetiologies. Quality of life and function returned to pre-prosthesis levels with discontinued use of prosthesis.

## Introduction

Limb loss is a devastating and debilitating condition that leads to dramatic changes in the lives of amputees. Reports from high-income countries (HICs) have documented that amputations negatively impact the quality of life (QOL ), posing significant physical and psychosocial challenges on amputees (Sinha, Van den Heuvel & Arokiasamy 2011). In HICs, the most common aetiologies of lower extremity amputation are peripheral vascular disease and diabetes, but in low- and middle-income countries (LMICs), the most common aetiologies include trauma, infection, diabetes and malignancy (Agu & Ojiaku 2016; Chalya et al. 2012; Gebreslassie, Gebreselassie & Esayas 2018; Grudziak et al. 2017; Loro & Franceschi 1999; Ogeng’o, Obimbo & King’ori 2009; Thanni & Tade 2007). In LMICs, traumatic injuries now cause more death and disability than malaria, tuberculosis and HIV combined (James et al. 2018) because of wartime conflicts and the increase in road traffic accidents associated with rapid urbanisation (Harkins, McGarry & Buis 2013). Concurrently, as populations in LMICs age, the impact of non-communicable diseases, such as obesity and diabetes, has grown (Hossain, Kawar & El Nahas 2007). The net effect of this changing health burden in LMICs is a growing number of amputees with severe disability and few resources allocated to manage their challenging condition (‘World Report on Disability’ n.d.). It is estimated that over 29 million individuals in resource-limited environments are in need of orthotic and prosthetic services (Harkins et al. 2013).

In HICs, treatment for limb loss focuses on the physical and psychosocial effects of amputation and usually includes the provision of a prosthesis to improve mobility (Wurdeman, Stevens & Campbell 2017). Prosthesis usage is associated with higher levels of employment, higher QOL and reduced secondary health issues (Pasquina, Carvalho & Sheehan 2015), although patients with dysvascular amputations report worse function than those with traumatic amputations (Amtmann et al. 2015). Compared to the robust literature on the impact of limb loss and benefits of prostheses in HICs, little has been done in LMICs, and much of the HIC-produced research is poorly applicable to the LMIC environment (Aluede et al. 2012; Harkins et al. 2013). Unique to LMIC prosthetic needs is the importance of affordability, durability and repairability (Wyss et al. 2015). Therefore, the selection of context-appropriate prostheses is critical to achieving the benefits of improved function, aesthetics and productivity associated with QOL.

Whilst some LMIC studies describe the aetiologies of lower-extremity amputation and lack of prosthetic and rehabilitation services (Agu & Ojiaku 2016; Chalya et al. 2012; Cummings 1996; Gebreslassie et al. 2018; Grudziak et al. 2017; Loro & Franceschi 1999; Maqsood et al. 2015; Ogeng’o et al. 2009; Thanni & Tade 2007), literature fails to address post-amputation determinants of QOL, function or impacts of prosthesis provision. Whilst the need for greater access to prosthetic services in LMICs is well established (Cummings 1996), the actual provision of these services has yet to meet those stated needs because of factors including prohibitive costs to both patients and institutions, lack of trained local prosthetics professionals and poor infrastructure for post-amputation care (Harkins et al. 2013; Ibrahim et al. 2019; Wyss et al. 2015). There remains a gap in the literature demonstrating the magnitude of QOL and functional benefits before and after the provision prostheses. Studies that further the understanding of the benefits of prostheses in LMICs will add needed weight to advocacy efforts for increased access to prosthetic services for amputees.

The objective of this study was to measure the impact of prostheses on QOL and function in transfemoral (TF) amputees in Tanzania. Prosthesis provision was hypothesised to improve QOL and function in TF amputees.

## Methods

We conducted a single-arm pre-post prospective cohort study enrolling TF amputees at Muhimbili Orthopaedic Institute (MOI) in Dar es Salaam, Tanzania.

### Study participants

**FIGURE 1:**
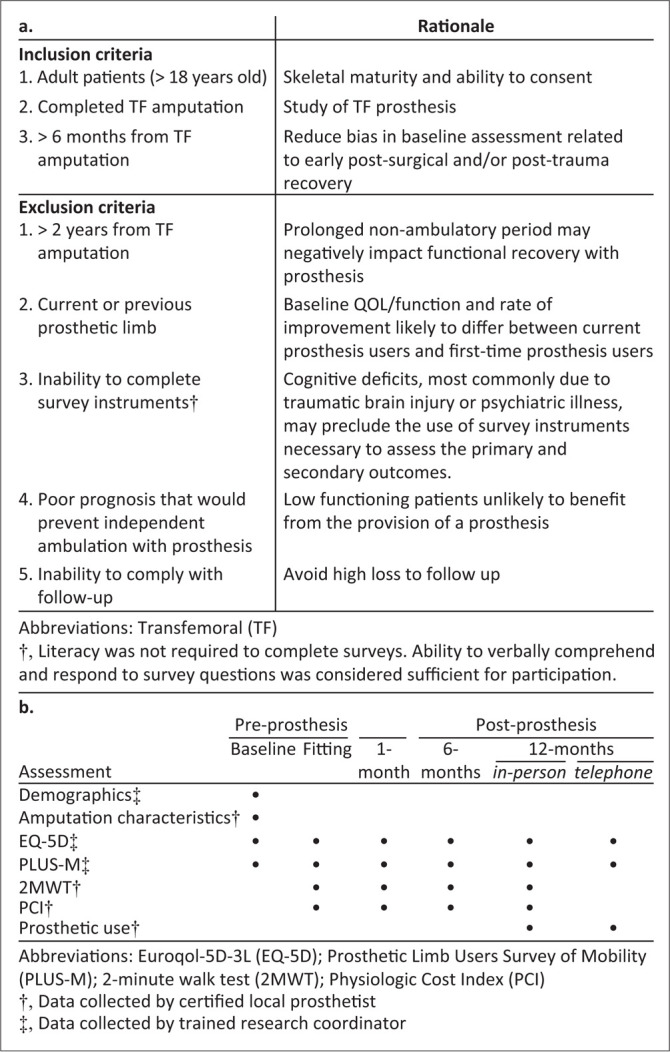
Eligibility criteria and schedule of data collection events: (a) the inclusion and exclusion criteria along with rationale used to generate the included cohort of patients and (b) the schedule of which assessments were performed at each timepoint throughout the study. The data collected at 12 months was dependent on whether the visit was conducted in-person or by telephone.

All patients presenting to MOI Prosthetics and Orthotics workshop with TF amputation were screened for eligibility between June 2017 and July 2018 (see Figure 1a for eligibility criteria). Written informed consent was obtained.

### Sample size

The study was powered to detect a difference in EuroQol-5D-3L (EQ-5D-3L) before and after the provision of a prosthesis. Power calculations were performed based on a pilot study of 21 TF amputees (Shaw et al. 2018) that reported a change in EQ-5D of 0.3 (standard deviation [SD]: 0.25) with prosthesis use. To achieve 90% power and a Bonferroni-corrected alpha of 0.0125 for four repeated measurements, the study required at least 10 patients. Assuming a loss to follow-up rate of 20%, the minimum enrolment was 13 amputees.

### Intervention

All patients received a definitive modular endoskeletal transfemoral prosthesis, which is typical for the region and within the technical capabilities of local providers. The prosthesis included Ottobock (Germany) socket materials: stockinette, carbon fibre and lamination resin, the LegWorks (Canada) All-Terrain Knee, Ortpar Ortopedi (Turkey) alignable components and Solid Ankle Cushion Heel (SACH) foot and local Tanzanian supplies: cosmetic foams, stockings and plaster of Paris. Suspension of the prosthesis was achieved by skin fit suction methods. A Silesian belt was added if needed. All components and materials were selected collaboratively with research partners and local providers. Prostheses were provided at no cost to study participants, and all fitting and fabrication were performed by certified local prosthetists. Gait training was performed by prosthetists during fitting and dynamic alignment, prior to application of the outer cosmetic foam. The quality of prosthetic fit and alignment was assessed via annual site visits by UCSF prosthetists.

### Study timeline

Participants were assessed at baseline before receiving prostheses and followed for 1 year after fitting at 1, 6 and 12 months. Quality of life and functional outcomes, including EQ-5D-3L, Prosthetic Limb Users Survey of Mobility 12-item short form (PLUS-M), 2-minute walk test (2MWT) and Physiologic Cost Index (PCI), were assessed and analysed for change over time (Figure 1b).

Patients who were unable to attend the 12-month follow-up visit in person were contacted by telephone, and only EQ-5D and PLUS-M data were collected.

### Baseline data

Basic demographic data including age, sex, employment, tobacco and alcohol use and estimated pre-amputation health-related QOL (HRQOL ) and indication for amputation were obtained at the initial visit.

### Patient-reported outcomes

EuroQol-5D and PLUS-M questionnaires were administered to assess HRQOL and mobility, respectively. The EQ-5D is a five-question validated, standardised, nondisease-specific instrument for describing and valuing HRQOL based on five dimensions: mobility, self-care, usual activities, pain or discomfort and anxiety or depression (eds. Szende, Oppe & Devlin 2007). The EQ-5D Swahili translation, a readily available and validated version, was converted to an index score ranging from −0.145 to 1 using weightings based on data from Zimbabwe (eds. Szende et al. 2007). The PLUS-M is a 12-question validated instrument used to measure mobility by assessing respondents’ perceived ability to carry out specific activities that require the use of both lower limbs (‘Prosthetic Limb Users Survey of Mobility [PLUS-M^TM^] Version 1.2 Short Forms Users Guide’ 2014). Prosthetic Limb Users Survey of Mobility was translated to Swahili using the method recommended by the instrument developer, which included both forward- and back-translation using professional translators. The survey responses were converted to an index score (T-score) ranging from 17.5 to 76.6 using the recommended algorithm (‘Prosthetic Limb Users Survey of Mobility [PLUS-M^TM^] Version 1.2 Short Forms Users Guide’ 2014). At the initial visit, patients were asked to recall EQ-5D and PLUS-M for the period prior to amputation, (pre-amputation) although these data were not included in statistical analyses to avoid recall bias. The questionnaires were also administered at the following timepoints: at the casting visit before the provision of the prosthesis (pre-prosthesis) and at 1, 6 and 12 month follow-up visits after prosthesis fitting.

### Functional outcomes

Function was assessed using 2MWT and PCI at pre-prosthesis and each subsequent visit. 2-Minute walk test and PCI are functional metrics to assess patients’ mobility as a function of the distance a patient can ambulate in 2 min, including changes in heart rate (HR) during activity (Guirao et al. 2017). Patients walked along a corridor marked every 1.5 m, and the total distance ambulated within 2 min was recorded. Time was measured using a stopwatch, and distance was measured according to the 1.5 m distance markings, to the nearest meter. Heart rate was measured and recorded before and after the 2MWT using an HR monitor (Polar FT7, Polar Electro, Kempele, Finland). Physiologic Cost Index has been used as a simple, indirect measure of oxygen cost during exercise and is defined as (Fredrickson, Ruff & Daly 2007):

$$\text{PCI}(\text{beats}/\text{m})=\frac{{\text{HR}}_{\text{steady state exercise}}(\text{beats}/\text{min})-{\text{HR}}_{\text{rest}}(\text{beats}/\text{min})}{\text{Walking speed}(\text{m}/\text{min})}$$ [Eqn 1]

All measurements completed prior to prosthesis provision were assessed with patients using only their preferred assistive devices.

### Data collection and statistical analysis

Data were collected by local research coordinators and certified prosthetists on laptop computers into Research Electronic Data Capture (REDCap), a secure, web-based software platform designed to support data capture, hosted at UCSF (Harris et al. 2009, 2019). Amputation characteristics were assessed by prosthetists. Baseline patient characteristics and EQ-5D, PLUS-M, 2MWT and PCI were collected by trained research coordinators.

De-identified data were exported to Stata 16.0 for analysis. One-way repeated measures Analysis of Variance (ANOVA) and post hoc pairwise comparison with Bonferroni correction were used for the analysis of continuous outcomes over time. For comparison between amputation etiology subgroups and prosthesis use subgroups, unpaired student’s *t*-test was used for continuous variables, and Fisher’s exact test was used for categorical variables and *p*-value of 0.05 was used for significance.

### Ethical considerations

The study was approved by the Ethical Review Board of the University of California, San Francisco (IRB#15-15804; Ref#244759), Holland Bloorview Kids Rehabilitation Hospital (REB#16-686) and the National Institute for Medical Research in Tanzania (Ref. NIMR/HQ/R.8a/Vol. IX/2122).

## Results

### Study population

**FIGURE 2:**
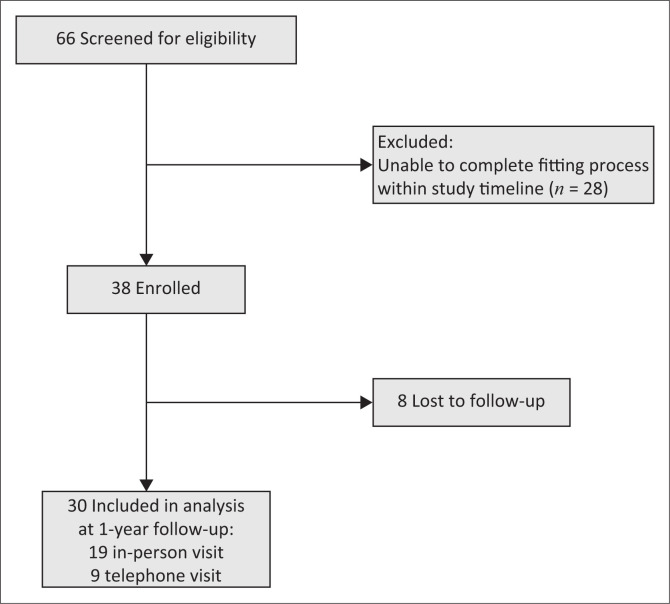
Flowchart demonstrating screening, enrolment and follow-up for study participants.

Of the 38 TF amputees enrolled, 30 (78.9%) had complete EQ-5D data at a minimum of 6 months after prosthesis fitting and were included in final data analysis (Figure 2, Table 2-A1). The mean age was 46 years (SD: 17.6), the meantime since amputation was 388 days (range 183–803) and the mean estimated EQ-5D before amputation was 1.00 (SD: 0.03). At 1 year after fitting, 20 (71%) patients reported using their prosthesis (Table 1).

**TABLE 1:** Patient demographic.

Factor	All patients				Amputation reason								*p*
					Non-vascular				Vascular				
	*N*	%	Mean (SD)	Mean (range)	*N*	%	Mean (SD)	Mean (range)	*N*	%	Mean (SD)	Mean (range)	
*N*	30	-	-	-	20	-	-	-	10	-	-	-	-
**Age (years)**	45.87	-	17.61	-	37.75	-	13.39	-	62.10	-	13.47	-	< 0.01*
Sex†	26	87	-	-	18	90	-	-	8	80	-	-	0.58
**Employed prior to amputation**	-	-	-	-	-	-	-	-	-	-	-	-	0.06
No	2	7	-	-	1	5	-	-	1	10	-	-	-
Formal employment	8	27	-	-	3	15	-	-	5	50	-	-	-
Informal employment	20	67	-	-	16	80	-	-	4	40	-	-	-
Employed since amputation‡	3	10	-	-	3	15	-	-	0	0	-	-	0.53
**Smoker**	-	-	-	-	-	-	-	-	-	-	-	-	0.03*
Current	1	3	-	-	1	5	-	-	0	0	-	-	-
Former	5	17	-	-	1	5	-	-	4	40	-	-	-
Never	24	80	-	-	18	90	-	-	6	60	-	-	-
Alcohol use§	6	20	-	-	2	10	-	-	4	40	-	-	0.14
**Amputation side**	-	-	-	-	-	-	-	-	-	-	-	-	0.14
Left	18	60	-	-	14	70	-	-	4	40	-	-	-
Right	12	40	-	-	6	30	-	-	6	60	-	-	-
Days from amputation to prosthesis fitting	387.79	-	-	183–803	412	-	-	195.97	304.80	-	-	138.98	0.13
Used assistive devices for ambulation	17	65	-	-	9	53	-	-	8	89	-	-	0.1
Diabetes¶	8	27	-	-	1	5	-	-	7	70	-	-	< 0.01*
Peripheral vascular disease¶	5	17	-	-	0	0	-	-	5	50	-	-	< 0.01*
Other comorbidities‡‡	4	13	-	-	2	10	-	-	2	20	-	-	0.58
Prosthesis use at 12 months§§	20	71	-	-	16	84	-	-	4	44	-	-	0.07
EQ-5D index prior to amputation	1.00	-	0.03	-	1	-	0	-	0.99	-	0.05	-	0.16
EQ-5D VAS prior to amputation	94.83	-	11.21	-	93.75	-	13.36	-	97	-	4.50	-	0.46

SD, standrad deviation; EQ-5D, EuroQol-5D; VAS, visual analog scale.

* , *p* ≤ 0.05.

† , Sex reported as number and % of population male.

‡ , Employment reported as % of population who are employed.

§ , Alcohol use reported as % of population who use alcohol.

¶ , Diabetes reported as % of population with diabetes.

†† , Peripheral vascular disease reported as % of population with peripheral vascular disease.

‡‡ , Includes: heart, lung and kidney disease and stroke.

§§ , Prosthesis use reported as % of population using prosthesis at 12 months.

We assessed the indications for amputation and found that 15 (50.0%) were because of trauma, seven (23.3%) diabetes, four (13.3%) tumour, three (10.0%) vascular disease and one (3.3%) chronic osteomyelitis. Patients who received TF amputation for trauma, tumour or infection were significantly younger (38 years, SD: 13.4) than patients who received TF amputation for diabetes or vascular disease (62 years, SD: 13.5; *p* < 0.01). The trauma, tumour and infection amputees, 20 (66.7%), were categorised as the ‘non-vascular’ subgroup and the diabetes and vascular disease amputees, 10 (33.3%), were categorised as the ‘vascular’ subgroup. There was no difference between the subgroups in estimated pre-amputation EQ-5D (*p* = 0.16).

### Patient-reported outcomes

**FIGURE 3:**
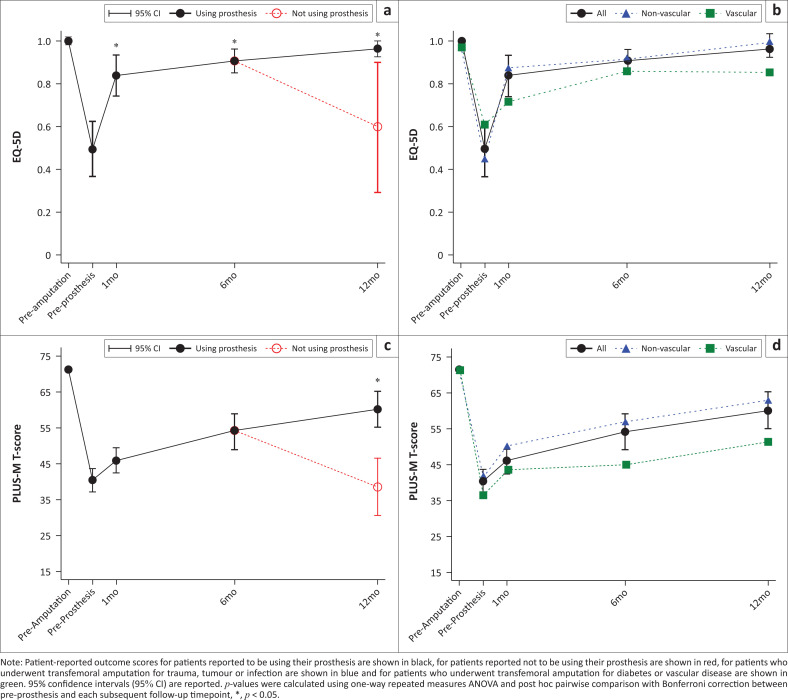
Patient-reported outcomes before amputation, at the casting visit before prosthesis fitting, 1, 6 and 12 months after prosthesis fitting: (a) EuroQol-5D (EQ-5D) health status scores before amputation (pre-amputation), at the casting visit before prosthesis fitting (pre-prosthesis) and at 1 month (1 mo), 6 months (6 mo) and 12 months (12 mo) follow-up after prosthesis fitting; (b) EQ-5D health status scores for patients separated by the reason for amputation at pre-amputation, pre-prosthesis, 1 mo, 6 mo and 12 mo for patients reported to be using their prosthesis; (c) Prosthetic Limb Users Survey of Mobility (PLUS-M) scores at pre-amputation, pre-prosthesis, 1 mo, 6 mo and 12 mo; (d) PLUS-M scores for patients separated by the reason for amputation at pre-amputation, pre-prosthesis, 1 mo, 6 mo, and, 12 mo for patients reported to be using their prosthesis.

EuroQol-5D was higher than pre-prosthesis baseline (0.50) at 1 month (0.84, *p <* 0.001), 6 months (0.91, *p* < 0.001) and 12 months (0.86, *p* < 0.001) after prosthesis fitting (repeated-measures ANOVA, *p* < 0.001; Figure 3a , Table 1-A1). At 12 months, -EQ-5D was higher in patients who reported using their prostheses (0.96) than for those who reported not using their prostheses (0.60, *p* < 0.001). At 12 months, EQ-5D in the non-vascular subgroup (0.99) was higher than the vascular subgroup (0.85, *p* < 0.001) (Figure 3b, Table 1-A1).

Prosthetic Limb Users Survey of Mobility was higher than pre-prosthesis baseline (39.94) at 6 months (54.04, *p* < 0.001) and 12 months (53.71, *p* < 0.001) after prosthesis fitting (repeated-measures ANOVA, *p* < 0.001; Figure 3c, Table 1-A1). At 12 months, PLUS-M scores were higher for patients who reported using their prosthesis (60.12) than for those who reported not using their prosthesis (38.48, *p* < 0.001). At 12 months, PLUS-M for the non-vascular subgroup (62.50) trended higher than for the vascular subgroup (51.2, *p* = 0.052) (Figure 3d, Table 1-A1).

### Functional outcomes

**FIGURE 4:**
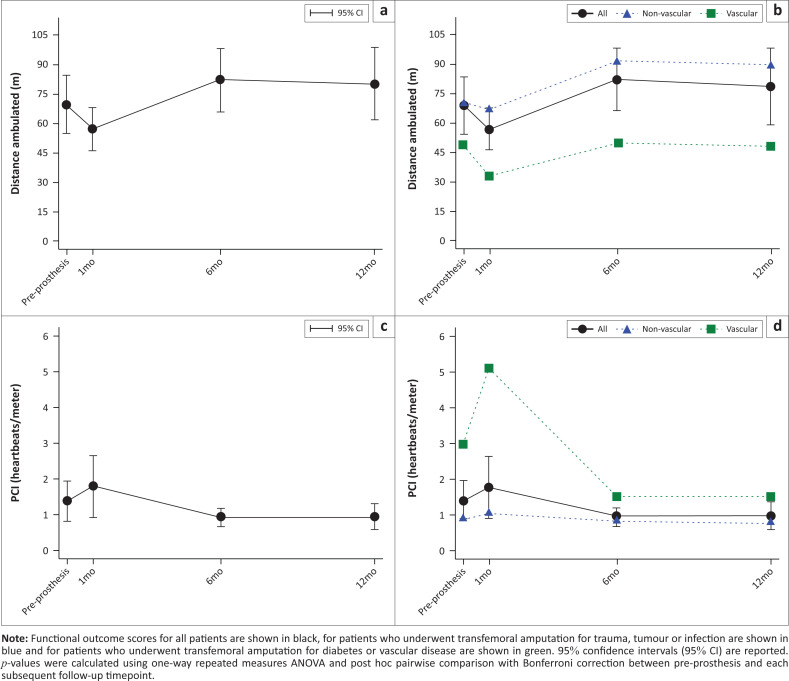
Functional outcomes before amputation, at the casting visit before prosthesis fitting, 1, 6 and 12 months after prosthesis fitting: (a) 2-minute walk test scores at the casting visit before prosthesis fitting (pre-prosthesis) and at 1 month (1 mo), 6 months (6 mo) and 12 months (12 mo) follow-up after prosthesis fitting; (b) 2-minute walk test scores for patients separated by the reason for amputation at pre-prosthesis, 1 mo, 6 mo and 12 mo; (c) Physiologic Cost Index (PCI) at pre-prosthesis, 1 mo, 6 mo and 12 mo and (d) PCI for patients separated by the reason for amputation at pre-prosthesis, 1 mo, 6 mo and 12 mo.

The distance ambulated, in meters, during the 2MWT increased after prosthesis fitting (repeated-measures ANOVA, *p* < 0.001), trending higher than pre-prosthesis baseline (68.26) at 6 months after prosthesis fitting (84.87, *p* = 0.059; Figure 4a, Table 1-A1). Distance ambulated by the non-vascular subgroup was higher than the vascular subgroup at 1 month (66.81, *p* = 0.018) and 6 months (91.47, *p* = 0.024) after prosthesis fitting (33.25 and 50.20, respectively) (Figure 4b, Table 1-A1).

Physiologic Cost Index was never significantly different from pre-prosthesis baseline (repeated-measures ANOVA *p* = 0.0623; Figure 4c, Table 1-A1). Physiologic Cost Index was significantly lower in the non-vascular subgroup than in the vascular subgroup at pre-prosthesis baseline (0.90 vs. 2.98, *p* = 0.011), 1 month (1.03 vs. 5.11, *p* < 0.001) and 6 months (0.82 vs. 1.53, *p* = 0.010) after prosthesis fitting (Figure 4d, Table 1-A1).

### Prosthesis use at 12 months after fitting

At 1 and 6 months after fitting, all patients reported using their prosthesis. At 12 months after fitting, 16 (84%) patients in the non-vascular subgroup reported using their prosthesis whilst just four (44%) patients in the vascular subgroup reported using their prosthesis, although this difference did not reach statistical significance (*p* = 0.068).

Of the three patients in the non-vascular subgroup who reported not using their prosthesis, two described having fully abandoned their prosthesis, whilst the third expressed interest in resuming use after recovery from an unrelated illness. In contrast, all five patients in the vascular subgroup who reported not using their prosthesis described having fully abandoned the prosthesis. Reasons provided for prosthesis abandonment in the vascular subgroup included socket loosening leading to poor fit and contralateral amputation leading to wheelchair use.

## Discussion

We prospectively followed 30 TF prosthesis recipients for 1 year after fitting to measure impacts of prostheses on QOL and function. We found, as hypothesised, that HRQOL and function improved significantly after prosthesis provision. Whilst data are limited on impacts of prostheses in LMICs, HIC studies have reported improvements in QOL , mobility and secondary health issues with prosthesis usage in lower limb amputees (Pasquina et al. 2015). Here, we demonstrate similarly that the provision of a prosthesis improves HRQOL , mobility and function in TF amputees in Tanzania. The demographics of the cohort captured are consistent with previously documented Tanzanian amputee populations (Chalya et al. 2012; Shaw et al. 2018).

Our study showed that prosthesis benefits were greater for non-vascular compared to vascular amputation aetiologies. The non-vascular subgroup was found to be younger, likely representing a healthier subset of TF amputees with greater potential to benefit from prosthetic rehabilitation. In contrast, patients with amputations because of diabetes and vascular disease tended to be older with concurrent medical issues, leading to less overall benefit. The findings of these subgroup analyses mirror those reported in HICs, where patients with dysvascular amputations were significantly older, with more comorbidities and worse functional status and QOL than patients with amputations because of trauma (Amtmann et al. 2015). Dysvascular lower limb amputees in HICs have also been documented to use their prostheses less than amputees with trauma-related amputations (Raichle et al. 2008). Whilst not statistically significant, our study similarly demonstrated a trend that vascular subgroup TF amputees had a higher rate of prosthesis abandonment. Nonetheless, these dysvascular patients still experienced significant improvements in HRQOL and function after receiving a prosthesis.

When compared to QOL benefits of health interventions in other medical fields, our study highlights the magnitude of the impact of amputation and subsequent prosthesis provision on HRQOL. Our results show that before receiving prostheses, TF amputees have EQ-5D scores of 0.48, a score notably worse than EQ-5D levels associated with other pathologies, such as 0.64 for chronic obstructive pulmonary disease or 0.51 for cerebral infarction (Zhou et al. 2018). To our knowledge, the QOL of an amputee without a prosthetic device has not been previously reported, particularly at the transfemoral level. We found the provision of a prosthesis dramatically improved this EQ-5D score, with a HRQOL increase of 0.37 amongst all patients and 0.58 in the non-vascular subgroup. These improvements are well above previously described minimal clinically important differences for EQ-5D of 0.03–0.36 for musculoskeletal disorders and 0.074 for non-disease-specific (Coretti, Ruggeri & McNamee 2014). In addition to the significant impacts on QOL , the impacts on patient-reported mobility found in this study are supported by similar findings from HICs. Lower limb prosthesis users in HICs reported a PLUS-M score of 50.3 (Hafner et al. 2017), similar to the value (53.71) for prosthesis users 1 year after prosthesis provision measured in this study.

There is limited literature available about 2MWT and PCI in lower limb amputees, and the existing studies report considerable variability. The few studies of 2MWT in TF amputees in HICs report distances ambulated ranging from 40 m (Brooks et al. 2001) up to 135 m (Gaunaurd et al. 2020), and normative 2MWT reference values for healthy individuals have been reported as 150 m – 217 m (Bohannon 2017). Our findings of over 80 m ambulated at 6 and 12 months after prosthesis fitting fall within published 2MWT ranges. Published PCI values of 0.23–0.42 for healthy individuals and 0.57 for TF amputees (Vllasolli et al. 2015) are considerably lower than those found in our study. These differences likely stem from methodologic variation as the published studies primarily use five-minute walking tests and report higher walking speeds than found in our study.

This study is limited by follow-up duration that represents a relatively short proportion of clinically relevant timeframe in the prosthesis life cycle. Whilst clinical improvements in the cohort stabilised by 6 months after prosthesis provision, questions related to known long-term prosthesis concerns in LMICs such as durability, structural failure, excessive wear and deterioration because of sunlight and other environmental exposures (Wyss et al. 2015) can only be answered after longer periods of observation. An additional limitation is the inability to quantify fit and alignment of the prosthesis or provide adequate longitudinal gait training through the duration of the study. Although gait training was done by local prosthetists during prosthesis fitting, participants did not undergo the formalised longitudinal outpatient physical therapy gait training as is the standard of care in HICs. Standardisation in the fitting process was achieved by providing additional training to local prosthetists, however, the standard of care in LMICs does not include standardised assessment of fit, alignment or gait training. This limitation underscores the robustness of the studied intervention in that provision of a prosthesis significantly improved QOL and function of amputees even in the absence of the prosthetic adjustments and rehabilitation that would be common in HICs.

We screened a larger number of patients than were ultimately included in the study because of the inability of some patients to complete the fitting process within the study timeline. Even with the provision of a prosthesis at no cost to patients, challenges related to inconsistent availability of locally sourced materials, a limited number of trained providers and the need for patients to travel to the prosthetic workshop for multiple fitting visits contributed to the observed inefficiency of the fitting process in this resource-limited environment. Considering the sample size needed to power the study and the limited resources available to extend the timeline of the study, the number of patients ultimately included in the study was considered sufficient. Of note, regression analysis was not performed, so results should be interpreted with the understanding that there may be additional factors that contribute to observed improvements in QOL and function.

As self-reported survey instruments, the EQ-5D and PLUS-M measurements are prone to subjectivity, although both have been extensively validated (Hafner et al. 2017; eds. Szende et al. 2007). Further, as participants were enrolled following amputation, prospective data were not available for pre-amputation baseline, so participants were asked to recall this state. In order to avoid recall bias, these recalled values were provided for reference but were not used in the outcome analysis. The use of the PLUS-M for amputees without prostheses has not yet been validated, so the values reported pre-prosthesis should be interpreted as such with respect to participants’ self-reported mobility.

Finally, the generalisability of this study is limited by the use of only one type of prosthesis that may or may not produce results similar to other transfemoral prostheses. The prosthesis used in this study was selected based on the experience of local providers as well as the availability of the components and materials via manufacturer distribution and non-governmental organisation (NGO) programmes. Thus, given the clinical improvements we observed, we believe it has the potential to be sustainably implemented broadly in low-resource settings. Further, our findings are consistent with the benefits of prosthetics measured in HICs (Amtmann et al. 2015), which suggests that these results may be broadly applicable.

## Conclusion

Our findings demonstrate that the provision of a prosthetic device to transfemoral amputees in an LMIC improves both HRQOL and function. To our knowledge, the magnitude of this impact has never before been quantified in resource-limited settings and will add needed data to advocacy efforts for prosthesis provision in overburdened health systems. Additional investigations of long-term outcomes and cost-effectiveness of the observed health benefits are needed to more strongly advocate for universal prosthesis provision in LMICs.

## Appendix 1

**TABLE 1-A1:** Patient-reported outcomes of health-related quality of life and mobility before amputation (pre-amputation), at the casting visit before prosthesis fitting (pre-prosthesis) and at 1 month (1 mo), 6 months (6 mo) and 12 months (12 mo) follow-up after prosthesis fitting.

Variable	Pre-amputation		Pre-prosthesis		1-month follow up		6-month follow up		12-month follow up		*p* (repeated-measures ANOVA)
	Mean	95% CI	Mean	95% CI	Mean	95% CI	Mean	95% CI	Mean	95% CI	
EQ-5D	1.00	0.99–1.01	0.50	0.37–0.62	0.84	0.74–0.93*	0.91	0.85–0.96*	0.86	0.76–0.96*	< 0.01**
PLUS-M T-score	71.10	70.50–71.71	39.94	36.80–43.08	45.85	42.38–49.33	54.04	49.07–59.01*	53.71	48.09–59.33*	< 0.01**
2MWT	-	-	68.26	51.86–84.67	57.37	46.29–68.45	84.87	66.93–102.81	80.54	57.02–104.06	< 0.01**
PCI	-	-	1.49	0.85–2.14	1.85	0.96–2.74	0.88	0.69–1.08	0.97	0.53–1.40	0.06

Note: Functional outcomes at pre-prosthesis, 1 mo, 6 mo and 12 mo. EuroQol-5D, PLUS-M, 2MWT and PCI are reported as mean and 95% confidence interval (CI). The *p-*values were calculated using one-way repeated measures ANOVA, **, *p* < 0.05, and post hoc pairwise comparison with Bonferroni correction between pre-prosthesis and each subsequent follow-up timepoint, *, *p* < 0.05.

EQ-5D, EuroQol-5D; PLUS-M, Prosthetic Limb Users Survey of Mobility; 2MWT, two-minute walk test; PCI, physiologic cost index; Analysis of Variance (ANOVA).

* ,*p* < 0.05;

** ,*p* < 0.05.

**TABLE 2-A1:** Data information for 12-month follow-up.

Variable	All patients		Amputation reason			
			Non-vascular		Vascular	
	*n*	%	*n*	%	*n*	%
*N*	28	-	**19**	**-**	**9**	-
**Prosthesis use**						
Using	20	71	16	84	4	44
Not using	8	29	3	16	5	56
**Type of visit**						
In-person	19	68	15	79	4	44
Telephone	9	32	4	21	5	56
EQ-5D data available	28	100	19	100	9	100
PLUS-M data available	28	100	19	100	9	100
2MTW data available	17	61	13	68	4	44
PCI data available	17	61	13	68	4	44

EQ-5D, EuroQol-5D; PLUS-M, Prosthetic Limb Users Survey of Mobility; 2MWT, two-minute walk test; PCI, physiologic cost index.

## References

[CIT0001] Agu, T.C. & Ojiaku, M.E., 2016, ‘The indications for major limb amputations: 8 years retrospective study in a private orthopaedic and trauma centre in the south-east Nigeria’, *Journal of Clinical Orthopaedics and Trauma* 7(4), 242–247. 10.1016/j.jcot.2016.03.006 27857497 PMC5106472

[CIT0002] Aluede, E.E., Phillips, J., Bleyer, J., Jergesen, H.E. & Coughlin, R., 2012, ‘Representation of developing countries in orthopaedic journals: A survey of four influential orthopaedic journals’, *Clinical Orthopaedics and Related Research* 470(8), 2313–2318. 10.1007/s11999-012-2377-5 22588702 PMC3392367

[CIT0003] Amtmann, D., Morgan, S.J., Kim, J. & Hafner, B.J., 2015, ‘Health-related profiles of people with lower limb loss’, *Archives of Physical Medicine and Rehabilitation* 96(8), 1474–1483. 10.1016/j.apmr.2015.03.024 25917819 PMC4519362

[CIT0004] Bohannon, R.W., 2017, ‘Normative reference values for the two-minute walk test derived by meta-analysis’, *Journal of Physical Therapy Science* 29(12), 2224–2227. 10.1589/jpts.29.2224 29643611 PMC5890237

[CIT0005] Brooks, D., Parsons, J., Hunter, J.P., Devlin, M. & Walker, J., 2001, ‘The 2-minute walk test as a measure of functional improvement in persons with lower limb amputation’, *Archives of Physical Medicine and Rehabilitation* 82(10), 1478–1483. 10.1053/apmr.2001.25153 11588757

[CIT0006] Chalya, P.L., Mabula, J.B., Dass, R.M., Ngayomela, I.H., Chandika, A.B., Mbelenge, N. et al., 2012, ‘Major limb amputations: A tertiary hospital experience in northwestern Tanzania’, *Journal of Orthopaedic Surgery and Research* 7, 18. 10.1186/1749-799X-7-18 22578187 PMC3413574

[CIT0007] Coretti, S., Ruggeri, M. & McNamee, P., 2014, ‘The minimum clinically important difference for EQ-5D index: A critical review’, *Expert Review of Pharmacoeconomics & Outcomes Research* 14(2), 221–233. 10.1586/14737167.2014.894462 24625040

[CIT0008] Cummings, D., 1996, ‘Prosthetics in the developing world: A review of the literature’, *Prosthetics and Orthotics International* 20(1), 51–60. 10.3109/03093649609164416 8740078

[CIT0009] Fredrickson, E., Ruff, R.L. & Daly, J.J., 2007, ‘Physiological Cost Index as a proxy measure for the oxygen cost of gait in stroke patients’, *Neurorehabilitation and Neural Repair* 21(5), 429–434. 10.1177/1545968307300400 17409390

[CIT0010] Gaunaurd, I., Kristal, A., Horn, A., Krueger, C., Muro, O., Rosenberg, A. et al., 2020, ‘The utility of the 2-Minute Walk Test as a measure of mobility in people with lower limb amputation’, *Archives of Physical Medicine and Rehabilitation* 101(7), 1183–1189. 10.1016/j.apmr.2020.03.007 32272105

[CIT0011] Gebreslassie, B., Gebreselassie, K. & Esayas, R., 2018, ‘Patterns and causes of amputation in Ayder Referral Hospital, Mekelle, Ethiopia: A three-year experience’, *Ethiopian Journal of Health Sciences* 28(1), 31–36. 10.4314/ejhs.v28i1.5 29622905 PMC5866287

[CIT0012] Grudziak, J., Gallaher, J., Banza, L., Cairns, B., Varela, C., Young, S. et al., 2017, ‘The effect of a surgery residency program and enhanced educational activities on trauma mortality in sub-Saharan Africa’, *World Journal of Surgery* 41(12), 3031–3037. 10.1007/s00268-017-4272-4 29018914

[CIT0013] Guirao, L., Samitier, C.B., Costea, M., Camos, J.M., Majo, M. & Pleguezuelos, E., 2017, ‘Improvement in walking abilities in transfemoral amputees with a distal weight bearing implant’, *Prosthetics and Orthotics International* 41(1), 26–32. 10.1177/0309364616633920 27052274

[CIT0014] Hafner, B.J., Gaunaurd, I.A., Morgan, S.J., Amtmann, D., Salem, R. & Gailey, R.S., 2017, ‘Construct validity of the Prosthetic Limb Users Survey of Mobility (PLUS-M) in adults with lower limb amputation’, *Archives of Physical Medicine and Rehabilitation* 98(2), 277–285. 10.1016/j.apmr.2016.07.026 27590443 PMC5276724

[CIT0015] Harkins, C.S., McGarry, A. & Buis, A., 2013, ‘Provision of prosthetic and orthotic services in low-income countries: A review of the literature’, *Prosthetics and Orthotics International* 37(5), 353–361. 10.1177/0309364612470963 23295896

[CIT0016] Harris, P.A., Taylor, R., Minor, B.L., Elliott, V., Fernandez, M., O’Neal, L. et al., 2009, ‘Research electronic data capture (REDCap) – A metadata-driven methodology and workflow process for providing translational research informatics support’, *Journal of Biomedical Informatics* 42(2), 377–381. 10.1016/j.jbi.2008.08.010 18929686 PMC2700030

[CIT0017] Harris, P.A., Taylor, R., Thielke, R., Payne, J., Gonzalez, N. & Conde, J.G., 2019, ‘The REDCap consortium: Building an international community of software platform partners’, *Journal of Biomedical Informatics* 95, 103208. 10.1016/j.jbi.2019.103208 31078660 PMC7254481

[CIT0018] Hossain, P., Kawar, B. & El Nahas, M., 2007, ‘Obesity and diabetes in the developing world – A growing challenge’, *New England Journal of Medicine* 356(3), 213–215. 10.1056/NEJMp068177 17229948

[CIT0019] Ibrahim, J.M., Serrano, S., Caldwell, A.M., Francisco, S., Eliezer, E.N. & Haonga, B.T., 2019, ‘Barriers to prosthetic devices at a Tanzanian hospital’, *East African Orthopaedic Journal* 13, 8.

[CIT0020] James, S.L., Abate, D., Abate, K.H., Abay, S.M., Abbafati, C., Abbasi, N. 2018, ‘Global, regional, and national incidence, prevalence, and years lived with disability for 354 diseases and injuries for 195 countries and territories, 1990–2017: A systematic analysis for the Global Burden of Disease Study 2017’, *The Lancet* 392(10159), 1789–1858. 10.1016/S0140-6736(18)32279-7 PMC6227754 30496104

[CIT0021] Loro, A. & Franceschi, F., 1999, ‘Prevalence and causal conditions for amputation surgery in the third world: Ten years experience at Dodoma Regional Hospital, Tanzania’, *Prosthetics and Orthotics International* 23(3), 217–224. 10.3109/03093649909071637 10890596

[CIT0022] Maqsood, M., Ali, N., Bhat, A., Bangroo, F.A., Dhanda, M.S. & Singh, R., 2015, ‘Current trends of major lower limb amputations at a tertiary care centre of Jammu, India’, *International Journal of Medical Science Research and Practice* 2(2), 77–80.

[CIT0023] Ogeng’o, J.A., Obimbo, M.M. & King’ori, J., 2009, ‘Pattern of limb amputation in a Kenyan rural hospital’, *International Orthopaedics* 33(5), 1449–1453. 10.1007/s00264-009-0810-5 19475408 PMC2899126

[CIT0024] Pasquina, C.P.F., Carvalho, A.J. & Sheehan, T.P., 2015, ‘Ethics in rehabilitation: Access to prosthetics and quality care following amputation’, *AMA Journal of Ethics* 17(6), 535–546. 10.1001/journalofethics.2015.17.6.stas1-1506 26075981

[CIT0025] Prosthetic Limb Users Survey of Mobility (PLUS-M™) Short Forms Users Guide Version 1.2. 2013, viewed 26 March 2020, from http://www.plus-m.org.

[CIT0026] Raichle, K.A., Hanley, M.A., Molton, I., Kadel, N.J., Campbell, K., Phelps, E. et al., 2008, ‘Prosthesis use in persons with lower- and upper-limb amputation’, *Journal of Rehabilitation Research and Development* 45(7), 961–972. 10.1682/jrrd.2007.09.0151 19165686 PMC2743731

[CIT0027] Shaw, J., Challa, S., Conway, D., Liu, M., Haonga, B., Eliezer, E. et al., 2018, ‘Quality of life and complications in lower limb amputees in Tanzania: Results from a pilot study’, *The Lancet Global Health* 6, S18. 10.1016/S2214-109X(18)30147-5

[CIT0028] Sinha, R., Van den Heuvel, W.J. & Arokiasamy, P., 2011, ‘Factors affecting quality of life in lower limb amputees’, *Prosthetics and Orthotics International* 35(1), 90–96. 10.1177/0309364610397087 21515894

[CIT0029] Szende, A., Oppe, M. & Devlin, N. (eds.), 2007, *EQ-5D value sets: Inventory, comparative review and user guide*, Springer Netherlands (EuroQol Group Monographs), Dordrecht. 10.1007/1-4020-5511-0

[CIT0030] Thanni, L.O.A. & Tade, A.O., 2007, ‘Extremity amputation in Nigeria – A review of indications and mortality’, *The Surgeon: Journal of the Royal Colleges of Surgeons of Edinburgh and Ireland* 5(4), 213–217. 10.1016/s1479-666x(07)80006-0 17849957

[CIT0031] Vllasolli, T.O., Orovcanec, N., Zafirova, B., Krasniqi, B., Murtezani, A., Krasniqi, V. et al., 2015, ‘Physiological Cost Index and comfort walking speed in two level lower limb amputees having no vascular disease’, *Acta Informatica Medica* 23(1), 12–17. 10.5455/aim.2015.23.12-17 25870485 PMC4384862

[CIT0032] World Health Organization (WHO), n.d., *World Report on Disability 2011*, p. 350, World Health Organization, Geneva.

[CIT0033] Wurdeman, S.R., Stevens, P.M. & Campbell, J.H., 2017, ‘Mobility Analysis of AmpuTees (MAAT I): Quality of life and satisfaction are strongly related to mobility for patients with a lower limb prosthesis’, *Prosthetics and Orthotics International*, viewed 04 February 2020, from https://journals.sagepub.com/doi/10.1177/0309364617736089. 10.1177/0309364617736089 PMC6146310 28990467

[CIT0034] Wyss, D., Lindsay, S., Cleghorn, W.L. & Andrysek, J., 2015, ‘Priorities in lower limb prosthetic service delivery based on an international survey of prosthetists in low- and high-income countries’, *Prosthetics and Orthotics International* 39(2), 102–111. 10.1177/0309364613513824 24335154

[CIT0035] Zhou, T., Guan, H., Yao, J., Xiong, X. & Ma, A., 2018, ‘The quality of life in Chinese population with chronic non-communicable diseases according to EQ-5D-3L: A systematic review’, *Quality of Life Research: An International Journal of Quality of Life Aspects of Treatment, Care and Rehabilitation* 27(11), 2799–2814. 10.1007/s11136-018-1928-y PMC6208588 29980994

